# Developmental disorders in children born to women with sickle cell disease: A report from the Boston Birth Cohort

**DOI:** 10.1002/jha2.478

**Published:** 2022-06-08

**Authors:** Martha Brucato, Eboni Lance, Sophie Lanzkron, Xiaobin Wang, Lydia H. Pecker

**Affiliations:** ^1^ Department of Internal Medicine/Pediatrics Johns Hopkins University School of Medicine Baltimore Maryland USA; ^2^ Department of Neurology and Developmental Medicine Kennedy Krieger Institute, Baltimore Maryland USA; ^3^ Division of Hematology Department of Medicine Johns Hopkins University School of Medicine Baltimore Maryland USA; ^4^ The Center on the Early Life Origins of Disease, Department of Population, Family and Reproductive Health Johns Hopkins Bloomberg School of Public Health Baltimore Maryland USA

## Abstract

Children exposed to maternal sickle cell disease (SCD) have many theoretical risks for developmental disorders, but little is known about long‐term outcomes for these children. We used the Boston Birth Cohort to compare developmental outcomes between children exposed to maternal SCD and matched, unexposed controls. Children with exposure to maternal SCD had increased risk of attention deficit hyperactivity disorder (OR 5.12, 95% CI 1.36–19.19, *p* = 0.02) and obesity (OR 2.74, 95% CI 1.10–6.87, *p* = 0.03). In utero and/or environmental exposures may help explain these findings. Further studies of outcomes of children born to women with SCD are needed.

**Abbreviations**
BBC: Boston Birth Cohort
IUGR: Intrauterine growth restriction
NDD: Neurodevelopmental disorder
SCD: Sickle cell disease

## 1 INTRODUCTION

In resource‐rich settings, over 95% of children with sickle cell disease (SCD) now survive into adulthood making reproductive outcomes increasingly important for affected individuals and their families [1]. Developmental outcomes of children exposed to maternal SCD who do not themselves have SCD are poorly defined. Many outcomes that occur among pregnant women with SCD are risks for developmental disorders in offspring. These include maternal anemia before the thirtieth week of pregnancy [2], inflammatory and abnormal immune activation, preeclampsia, intrauterine growth restriction (IUGR), preterm birth, low birth weight, maternal history of fetal demise [3], and chronic or intermittent intrauterine opioid exposure. These are common complications of SCD pregnancy [4], but little is known about long‐term developmental outcomes for children with a history of in utero exposure to maternal SCD [5].

Identifying whether children born to women with SCD have different developmental outcomes than children born to unaffected mothers requires a dataset that links maternal and child health data and a rigorously matched control group. Ideally this kind of data would be included in a sickle cell registry, but long‐term outcomes of offspring born to people with SCD are not yet captured [5]. The Boston Birth Cohort (BBC) is a well described, prospective birth cohort established in 1998 at the Boston Medical Center that recruits pre‐term and full‐term mother–baby dyads [6]. BBC data are collected through structured interviews at enrollment and by electronic medical record (EMR) review and include maternal preconception medical conditions, pregnancy complications and exposures, and child development outcomes. Women with SCD and their offspring in this cohort can be identified in this cohort.

This study's purpose was to use the BBC to compare developmental outcomes of children born to mothers with SCD to matched controls.

## 2 METHODS

The BBC is described [7]. It is a prospective birth cohort initially developed to investigate preconception and prenatal determinants of preterm birth. Dyads are recruited at delivery. Exclusion criteria are: multiple gestations, infants with chromosomal abnormalities or major birth defects, infants conceived through in vitro fertilization, and preterm delivery due to traumatic injury. The subset of children whose care continues at the Boston Medical Center has developmental outcomes captured in their EMR and remain linked to their birthing parent. Using EMR data for mothers and children from outpatient and emergency room encounters between October 1, 2003 and September 31, 2015, we identified all women with ICD‐9‐CM codes for SCD (282.60‐64, 282.68, 282.69, 282.41, 282.42). In anticipation of small numbers, we first liberally captured those with one or more SCD ICD‐9‐CM code (282.60‐64, 282.68, 282.69, 282.41, 282.42) [8] and then more conservatively identified those with two or more SCD ICD‐9‐CM codes. Comparison of characteristics of mothers and children identified with only one SCD code and ≥2 SCD codes identified no between‐group differences, so we included SCD dyads identified with at least one maternal SCD ICD‐9‐CM code (Tables S1 and S2). SCD is an independent risk factor for neurodevelopmental disorders (NDD), so dyads in which mother and child had SCD were excluded [9].

Statistical analyses were performed in R‐3.3.x. Using the R package MatchIt, dyad cases were matched to unaffected controls in a 1:3 ratio using propensity score analysis that matched cases and controls by probability of exposure to maternal SCD, conditional on race, marital status, education, and child's year of birth (Supporting figure). Logistic regression was first performed using the R *glm()* function. We calculated the log odds of maternal SCD status as a function of race, marital status, education, and child's year of birth and obtained fitted probabilities for all subjects. Matching was then performed to a case's nearest neighbor using the R package MatchIt. Child's year of birth was used for matching to account for secular trends since BBC data were collected between 2003 and 2015. We excluded women with sickle cell trait from the control group. Developmental outcomes identified by ICD‐9‐CM codes for NDD included delayed milestones, other speech and language delay, attention‐deficit disorder with hyperactivity (ADHD) and unspecified development delay (783.42, 315.39, 314.01, 315.9) and for physical development disorders including abnormal loss of weight, obesity, and failure to thrive (783.21, 278.00, 783.41). We used logistic regression to test the association between disorders of growth and development and maternal SCD exposure.

The Boston Medical Center and Johns Hopkins Bloomberg School of Public Health Institutional Review Boards approved this study. Participant informed consent was obtained per the Boston Medical Center Institutional Review Board protocol.

## 3 RESULTS

Among 5,972 BBC mothers, there were 2,992 children with available data. Among these mother–child dyads, 48 women had at least one ICD‐9‐CM code for SCD. Two mother–child dyads were excluded from further analysis because the child had SCD. Six mother–child dyads had either completely or partially missing demographic data and were excluded. Thus, the final analysis included 40 dyad cases and 120 controls, described in Table 1. Demographic characteristics of dyads with maternal SCD (*N* = 40) included self‐identification as 42.5% Black and 37.5% Haitian with a mean age 29.5 ± 6.6 years. Among children born to women with SCD, mean gestational age at birth was 38.77 weeks and birth weight for most (85%, *N* = 34/40) was > 2500 g. Gestational age at birth and birth weight were not significantly different from controls.

**TABLE 1:** Characteristics of exposed cases and unexposed controls matched on probability of exposure to maternal sickle cell disease, conditional on race, marital status, education, and child's year of birth

	Children exposed to maternal SCD (*n* = 40)	Unexposed controls (*n* = 120)	*p*
Maternal age, *M* (SD)	29.50 (6.58)	28.58 (6.46)	0.441
Child year of birth, *M* (SD)	2008.70 (2.08)	2008.70 (2.12)	1
Gestational age, mean (SD)	38.77 (1.75)	37.71 (3.58)	0.075
Education, *n* (%)			0.541
Elementary school	1 (2.5)	0 (0.0)	
Secondary school	7 (17.5)	20 (16.7)	
High school/GED	17 (42.5)	51 (42.5)	
Some college	9 (22.5)	30 (25.0)	
College degree and above	6 (15.0)	19 (15.8)	
Marital status, *n* (%)			0.851
Married	15 (37.5)	40 (33.3)	
Divorced	2 (5.0)	5 (4.2)	
Single	23 (57.5)	75 (62.5)	
Race or ethnicity, *n* (%)			0.997
Black	17 (42.5)	52 (43.3)	
White	1 (2.5)	3 (2.5)	
Hispanic	4 (10.0)	13 (10.8)	
Haitian	15 (37.5)	45 (37.5)	
Other	3 (7.5)	7 (5.8)	
Maternal smoking, *n* (%)			0.265
Never	37 (94.9)	101 (84.9)	
Some	1 (2.6)	9 (7.6)	
Continuous	1 (2.6)	9 (7.6)	
Child sex, *n* (%)			0.713
Female	21 (52.5)	69 (57.5)	
Male	19 (47.5)	51 (42.5)	
Birth weight, *n* (%)			0.452
>2500 g	34 (85.0)	91 (77.8)	
<2500 g	6 (15.0)	26 (22.2)	

Developmental outcomes in children exposed to maternal SCD compared to matched controls are shown in Table 2. NDD occurred in 38% of children born to women with SCD. Those with NDD had delayed milestones (15% prevalence overall), other developmental speech or language delay (18%), ADHD (15%), and unspecified delay in development (13%). The risk of ADHD was increased in children exposed to maternal SCD (OR 5.12, 95% CI 1.36–19.19, *p* = 0.02). A disorder of physical development occurred in 58% of exposed children. Exposed children had an increased risk of obesity (OR 2.74, 95% CI 1.10–6.87, *p* = 0.03) and decreased risk of abnormal weight loss (OR 0.44, 95% CI 0.21–0.93, *p* = 0.03).

**TABLE 2:** Risk of physical and cognitive developmental diagnoses in cases exposed to maternal SCD compared to unexposed, matched controls

	Cases (*n* = 40)	Controls (*n* = 120)	OR (95% CI)	*p*‐Value
Physical development				
Any abnormality	23	79	0.70 (0.34–1.46)	0.34
Abnormal loss of weight	15	69	0.44 (0.21–0.93)	**0.03**
Obesity	10	13	2.74 (1.10–6.87)	**0.03**
Failure to thrive	6	18	1.0 (0.37–2.72)	1
Neurodevelopment				
Any abnormality	15	55	0.71 (0.34–1.48)	0.36
Delayed milestones	6	34	0.44 (0.17–1.16)	0.10
Other developmental speech or language delay	7	38	0.46 (0.19–1.17)	0.11
Attention deficit disorder with hyperactivity	6	4	5.12 (1.36–19.19)	**0.02**
Unspecified delay in development	5	25	0.54 (0.19–1.53)	0.25

Significant associations (*p* < 0.05) are bolded.

## 4 DISCUSSION

Few studies report long‐term outcomes of children without SCD with in utero exposure to SCD. Here, children exposed to maternal SCD had higher odds of ADHD and obesity compared to children of matched controls. Both findings require additional study in larger cohorts. The overall NDD prevalence in this subset of the BBC (44%) is higher than a nationally representative sample [10].

ADHD diagnosis was increased in children exposed to maternal SCD. The mechanisms that mediate this finding are not defined. Children who have SCD are at increased risk for ADHD and behavioral issues in childhood [9], but little research identifies ADHD risk among children without SCD who are exposed to maternal SCD. Maternal SCD may change the in utero environment and affect fetal neurodevelopment. Maternal anemia and inflammation are associated with childhood ADHD [2, 11]. Maternal chronic illness is also associated with developmental disorders. The increased ADHD risk observed may reflect social and/or environmental consequences of having a parent with a chronic disease [12]. Children born to mothers with SCD may be more likely to be exposed to pain medications such as paracetamol or opioids in utero. These analgesics are associated with increased risk of ADHD in the larger BBC cohort [7, 13].

Children exposed to maternal SCD also had increased risk of obesity and decreased risk of abnormal weight loss compared to controls. Unexamined factors that increase obesity risk in this population may help explain why children were less likely to be diagnosed with abnormal weight loss. Although IUGR may be a risk factor for obesity [3], this outcome alone would not explain our findings as cases and matched controls had equal proportion of infants with low birth weight (Table 1). One plausible explanation for increased risk of obesity in children exposed to maternal SCD is that individuals with SCD are hypermetabolic, and children born to mothers with SCD may grow up in a calorie rich food environment [14]. Ultimately, this observational study cannot identify causal factors of obesity risk in children born to women with SCD, and more research is needed.

Suggestive differences in neurodevelopment and physical development were identified in this cohort between children exposed to maternal SCD and matched controls. Understanding the extent to which in utero exposures constitute risks for future child development is important because some exposures are modifiable for women with SCD. For example, if maternal anemia or opioid use do constitute risks for ADHD in this population, interventions like chronic red cell transfusion therapy can be used to prevent fetal exposure to maternal anemia [15]. Transfusion also helps reduce pain during pregnancy and might help limit opioid exposure.

There are limitations to this study. The study sample size is small and our conclusions cannot be definitive. Larger studies are required to validate the association seen here between maternal SCD and abnormal child development. We present data from mothers with SCD identified by at least one ICD‐9‐CM code for SCD. Ideally, individuals with SCD are identified by multiple SCD‐related ICD‐9‐CM codes [8]. Reliance on ICD‐9 codes may lead to misclassification error. This study also has several strengths. It leverages a unique resource to obtain preliminary data about unaffected children exposed to maternal SCD, a population about whom very little is known. We used propensity score matching for control subjects and match for race alongside variables that address social context. Even if SCD‐specific registries are updated to contain developmental outcomes for offspring, meaningful comparator groups without maternal SCD exposure will be necessary.

This study may serve as a springboard for future studies of women with SCD and their unaffected offspring. This information would be relevant to hematologists caring for pregnant women with sickle cell disease and pediatricians caring for their children. These studies are needed to define the physical and neurodevelopmental risks for this vulnerable and understudied population of women and children.

## CONFLICT OF INTEREST

Martha Brucato, Eboni Lance, and Xiaobin Wang have no conflicts of interest to declare. Lydia H. Pecker reports a consultancy for Forma Therapeutics outside the submitted work. Sophie Lanzkron received research funds to her institution from Pfizer, Ironwood, and Global Blood Therapeutics outside the submitted work.

## ETHICS STATEMENT

The Boston Medical Center and Johns Hopkins Bloomberg School of Public Health Institutional Review Boards approved this study. Participant informed consent was obtained per the Boston Medical Center Institutional Review Board protocol.

## Supporting information


**Table S1** Frequency of diagnoses related to physical and cognitive development in children born to mothers with SCD using liberal and stringent diagnostic criteria for SCD.
**Table S2** Characteristics of women with SCD identified by strict criteria of 2 or more SCD diagnoses (*n* = 19) compared to the additional women identified by using liberal criteria of only 1 SCD diagnosis (*n* = 21)

## Data Availability

The data that support the findings of this study are available from Dr. Xiaobin Wang upon reasonable request.
