# Supplementary material for: Developmental disorders in children born to women with sickle cell disease: A report from the Boston Birth Cohort
Source: EJHaem. 2022 Jun 8;3(3):894–8. doi: 10.1002/jha2.478 (PMC9421989; doi:10.1002/jha2.478)

**Supplemental Table 1**

Frequency of diagnoses related to physical and cognitive development in children born to mothers with SCD using liberal and stringent diagnostic criteria for SCD.

|  | **Maternal SCD by >=1 ICD-9-CM code**  **N=40** **N (%)** | **Maternal SCD by >=2 ICD-9-CM code** **N=19** **N (%)** |
| --- | --- | --- |
| **Physical development** | | |
| Any abnormality | 23 (57.5) | 10 (53) |
| Abnormal loss of weight | 15 (37.5) | 4 (21) |
| Obesity | 10 (25) | 6 (32) |
| Failure to thrive | 6 (15) | 3 (16) |
| **Neurodevelopment** | | |
| Any abnormality | 15 (37.5) | 8 (42) |
| Delayed Milestones | 6 (15) | 3 (16) |
| Other developmental speech or language delay | 7 (17.5) | 2 (11) |
| Attention deficit disorder with hyperactivity | 6 (15) | 3 (16) |
| Unspecified delay in development | 5 (12.5) | 2 (11) |

**Supplemental Table 2:** Characteristics of women with SCD identified by strict criteria of 2 or more SCD diagnoses (n=19) compared to the additional women identified by using liberal criteria of only 1 SCD diagnosis (n=21).

|  | **Maternal SCD by only 1 ICD-9-CM code**  **(n=21)** | **Maternal SCD by >=2 ICD-9-CM code**  **(n=19)** | **p** |
| --- | --- | --- | --- |
| Maternal age, M (SD) | 29.55 (7.93) | 29.45 (4.91) | 0.962 |
| Child year of birth, M (SD) | 2008.81 (2.29) | 2008.58 (1.87) | 0.731 |
| Gestational age, mean (SD) | 38.86 (1.80) | 38.68 (1.75) | 0.75 |
| Education, n (%) | | | 0.103 |
| Elementary school | 0 (0.0) | 1 (5.3) |  |
| Secondary school | 5 (23.8) | 2 (10.5) |  |
| High school/GED | 9 (42.9) | 8 (42.1) |  |
| Some college | 2 (9.5) | 7 (36.8) |  |
| College degree and above | 5 (23.8) | 1 (5.3) |  |
| Marital status, n (%) | | | 0.279 |
| Married | 9 (42.9) | 6 (31.6) |  |
| Divorced | 0 (0.0) | 2 (10.5) |  |
| Single | 12 (57.1) | 11 (57.9) |  |
| Race or Ethnicity, n (%) | | | 0.251 |
| Black | 8 (38.1) | 9 (47.4) |  |
| White | 1 (4.8) | 0 (0.0) |  |
| Hispanic | 4 (19.0) | 0 (0.0) |  |
| Haitian | 7 (33.3) | 8 (42.1) |  |
| Other | 1 (4.8) | 2 (10.5) |  |
| Maternal smoking, n (%) | | | 0.367 |
| Never | 19 (95.0) | 18 (94.7) |  |
| Some | 1 (5.0) | 0 (0.0) |  |
| Continuous | 0 (0.0) | 1 (5.3) |  |
| Child sex, n (%) | | | 0.739 |
| Female | 10 (47.6) | 11 (57.9) |  |
| Male | 11 (52.4) | 8 (42.1) |  |
| Birth weight, n (%) | | | 1 |
| >2500 grams | 18 (85.7) | 16 (84.2) |  |
| <2500 grams | 3 (14.3) | 3 (15.8) |  |

**Supplemental Figure**


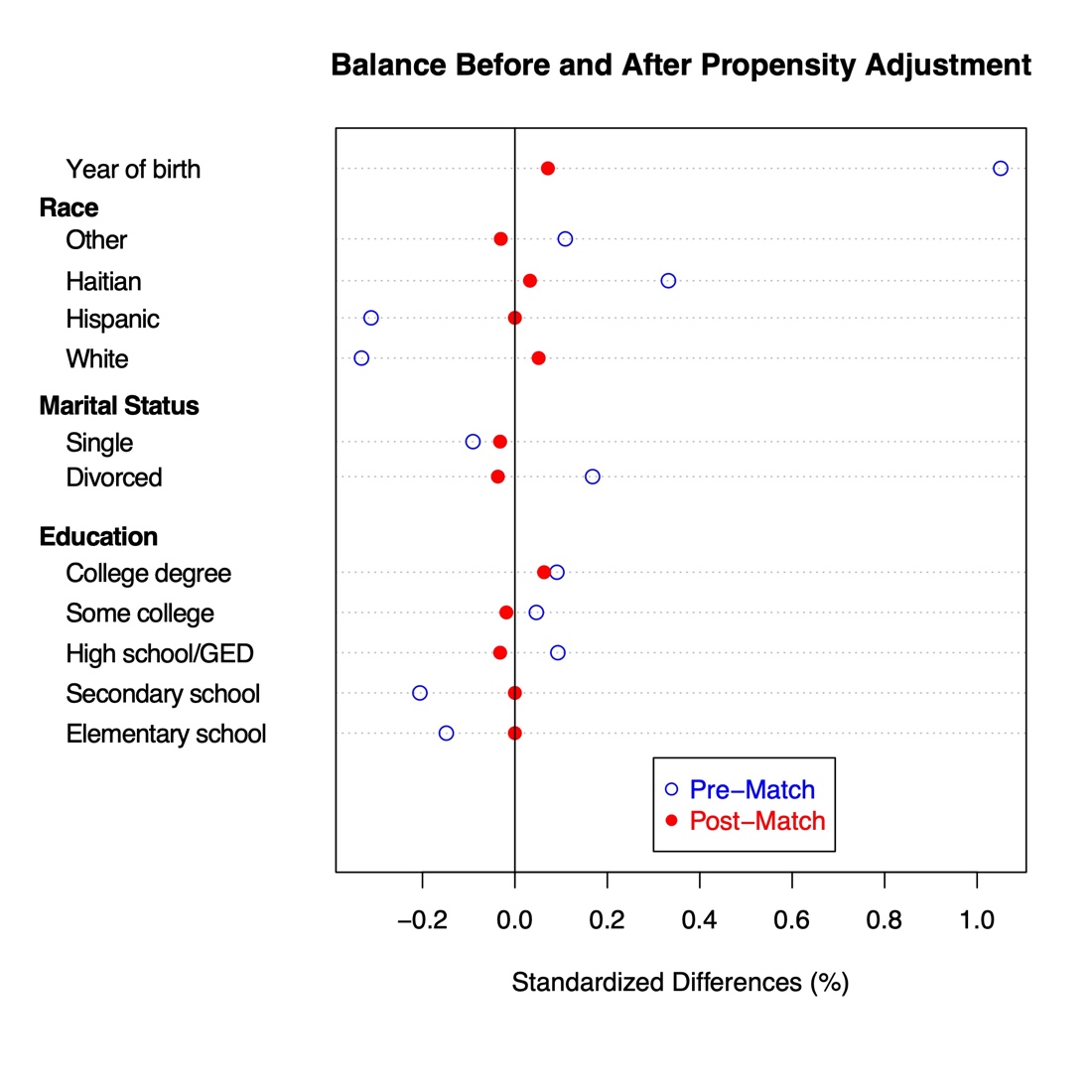

Supplement: Supplementary file 1 — Table S1 Frequency of diagnoses related to physical and cognitive development in children born to mothers with SCD using liberal and stringent diagnostic criteria for SCD. Table S2 Characteristics of women with SCD identified by strict criteria of 2 or more SCD diagnoses (n = 19) compared to the additional women identified by using liberal criteria of only 1 SCD diagnosis (n = 21) [file JHA2-3-894-s001.docx]
